# Establishing a high sensitivity detection method for SARS-CoV-2 IgM/IgG and developing a clinical application of this method

**DOI:** 10.1080/22221751.2020.1811161

**Published:** 2020-09-18

**Authors:** Chunyan Zhang, Lei Zhou, Hao Liu, Sibing Zhang, Yaping Tian, Junli Huo, Fei Li, Yao Zhang, Bo Wei, Dan Xu, Jinwei Hu, Jiayi Wang, Yuxuan Cheng, Wenjie Shi, Xiuli Xu, Jianping Zhou, Peipei Sang, Xudong Tan, Weiwei Wang, Minjie Zhang, Bin Wang, Yujun Zhou, Kan Zhang, Kunlun He

**Affiliations:** a Chinese PLA General Hospital, Beijing, People’s Republic of China; b Wuhan Huoshenshan Hospital, Wuhan, People's Republic of China; c Xijing Hospital of Air Force Medical University of PLA, Xi’an, People’s Republic of China; d The Second Affiliated Hospital of Naval Medical University, Shanghai, People’s Republic of China; e First Hospital of Changsha, Changsha, People’s Republic of China; f Beijing Diagreat Biotechnology Co., Ltd., Beijing, People’s Republic of China

**Keywords:** SARS-CoV-2 antibody, multi-epitopes fusion protein, time-resolved fluorescence immunoassay, disease evaluation, prognosis

## Abstract

COVID-19 is caused by SARS-CoV-2 infection and was initially discovered in Wuhan. This outbreak quickly spread all over China and then to more than 20 other countries. SARS-CoV-2 fluorescent microsphere immunochromatographic test strips were prepared by the combination of time-resolved fluorescence immunoassay with a lateral flow assay. The analytical performance and clinical evaluation of this testing method was done and the clinical significance of the testing method was verified. The LLOD of SARS-CoV-2 antibody IgG and IgM was 0.121U/L and 0.366U/L. The specificity of IgM and IgG strips in healthy people and in patients with non-COVID-19 disease was 94%, 96.72% and 95.50%, 99.49%, respectively; and sensitivity of IgM and IgG strips for patients during treatment and follow-up was 63.02%, 37.61% and 87.28%, 90.17%, respectively. The SARS-CoV-2 antibody test strip can provide rapid, flexible and accurate testing, and is able to meet the clinical requirement for rapid on-site testing of virus. The ability to detect IgM and IgG provided a significant benefit for the detection and prediction of clinical course with COVID-19 patients.

## Introduction

Severe Acute Respiratory Syndrome Coronavirus 2 (SARS-CoV-2), was discovered following an outbreak of viral pneumonia (COVID-19) cases in Wuhan in 2019 [1]. This outbreak quickly spread all over China and then to more than 20 other countries [2]. Coronavirus is a large family of viruses whose members are known to cause fever, Middle East respiratory syndrome (MERS), severe acute respiratory syndrome (SARS) and other severe diseases [3]. SARS-CoV-2 is a new Class VII coronavirus strain, which previously was not known to cause infection in human.

In general, there is an incubation period of 3∼14 days (up to 24 days as reported for some individual cases) after infections with SARS-CoV-2; moreover, and there may be no clinical symptoms during this incubation period [4]. Evidences at present show that infected persons are infectious during this incubation period, which has increased the difficulty of preventing spread of virus [5]. Clinical symptoms of COVID-19 are mainly manifested by fever, cough, weakness and other non-specific symptoms, which are hard to be distinguished from those symptoms of the common cold. Thus, the detection of infection by SARS-CoV-2 requires laboratory testing.

At present, the diagnosis of COVID-19 is based on detection of virus-specific gene sequence as determined by polymerase chain reaction (PCR) or nucleic acid sequencing [6]. However, nucleic acid testing requires special molecular testing laboratory (which requires 4 areas which are independent of each other in physical space) with supporting equipment and professional operating personnel (must be certified). Moreover, the test process is complex to operate and takes several hours. The molecular test procedure can generate aerosol pollution (amplicons), thus resulting in false-positive test results. There may also be false-negative test results due to inappropriate sampling. Therefore, molecular testing is not well suited for large-scale nucleic acid testing on site.

Immunological diagnosis of COVID-19 is mainly achieved through testing specific antibody IgM and IgG responses after human infection with SARS-CoV-2 and is based on antigen–antibody capture-methods. Such methods include lateral flow assays and provide the advantages of easy operation, quick test results, no need of a special laboratory site with (complex) instruments, and high sensitivity and specificity, and is suitable for carrying out large-scale SARS-CoV-2 infection/screening as point-of-care sites [7]. Several of the main methods of immunological diagnosis include colloidal gold method, ELISA and lateral flow assay. These tests detect virus-specific IgM and IgG antibody in blood and belong to indirect diagnostic methods. These test assays often use antigen and anti-human immunoglobulin secondary antibody to construct the test system. Many diagnosis methods of SARS-CoV-2 use either nucleocapsid protein (N Protein) or spike protein (S protein) alone as antigen. According to the reports of SARS antibody detection, the antibodies produced by human body mainly target N and S proteins, so using N and S proteins as antigens simultaneously may be able to detect more antibodies, thus improving the detection sensitivity. In addition, the SARS-CoV-2 N protein is easy to aggregate and unstable (unpublished data), which may be due to the high isoelectric point, which will also cause non-specific adsorption during the detection, resulting in false-positive results. Therefore, the antigen used for detection is critical to the sensitivity and specificity of the detection system.

Research for SARS in 2003 [8] have shown that combining antibody detecting with existing RT–PCR, the laboratory confirmation for SARS-CoV infection was greatly enhanced by 24.1%, from 48.1% (RT–PCR alone) to 72.2%. Meanwhile, several studies have proven that IgM and IgG antibodies against SARS-CoV can be detected within 2 weeks after illness onset and increased to detectable levels at the third week of illness due to SARS-CoV [9,10].

However, currently that has been few publications available that describe antibody testing against SARS-CoV-2. Therefore, in this study we have focused on producing a multi-epitope fusion protein-based antibody assay using the SARS-CoV-2 N and S protein in order to establish a SARS-CoV-2 antibody detecting method.

Based on the process of SARS-CoV-2 infection and the production of specific antibody responses, a diagnostic IgG and IgM detection assay would be the most useful method to diagnosis the occurrence of COVID-19 and development of pulmonary disease.

## Materials and methods

### Patients

A total of 1722 samples obtained from a total of 1,548 COVID-19 patients and non-COVID-19 controls from December 2019 to March 2020 were collected for the present study. These patients included 164 COVID-19 hospitalized patients, 234 COVID-19 follow-up patients, 154 suspected COVID-19 patients, 396 patients with other diseases, and 600 healthy controls. Among the COVID-19 hospitalized patients, 94 patients were continuously monitored for IgM and IgG antibodies (268 tests). Therefore, a total of 338 samples were obtained from the 164 patients.

### Reagents and equipment

The 1-ethyl-(3-Dimethyl aminopropyl) carbodiimide (EDC) was purchased from Shanghai Aladdin Bio-Chem. The NC membrane: CN140, Sartorious Company (Germany). Time-resolved fluorescent microsphere (200 nm): Nanjing Microdetection Bio-Tech Co., Ltd. The goat anti-human IgM µ chain antibody, goat anti-human IgG Fc antibody, goat anti-chicken IgY and chicken IgY were purchased from Luoyang Bai Aotong Experimental Materials Center.

Spot-spray system: Bio-Dot XYZ-3060, BIO-DOT (USA). High-speed refrigerated centrifuge: Hunan Changsha Xiangyi Centrifuge Instrument Co., Ltd. Magnetic stirrer: HJ-6, Jiangsu Jintan Giant Instrument Factory. Vacuum drying oven: DHG-9420A, Shanghai Shanzhi Instrument Equipment Co., Ltd. Fluorescent chromatography detector: Jinhaofeng Bio-tech Co., Ltd.

## Methods

### Preparation of the fusion recombinant protein of SARS-CoV-2 multi-dominant epitopes

**Figure 1.**
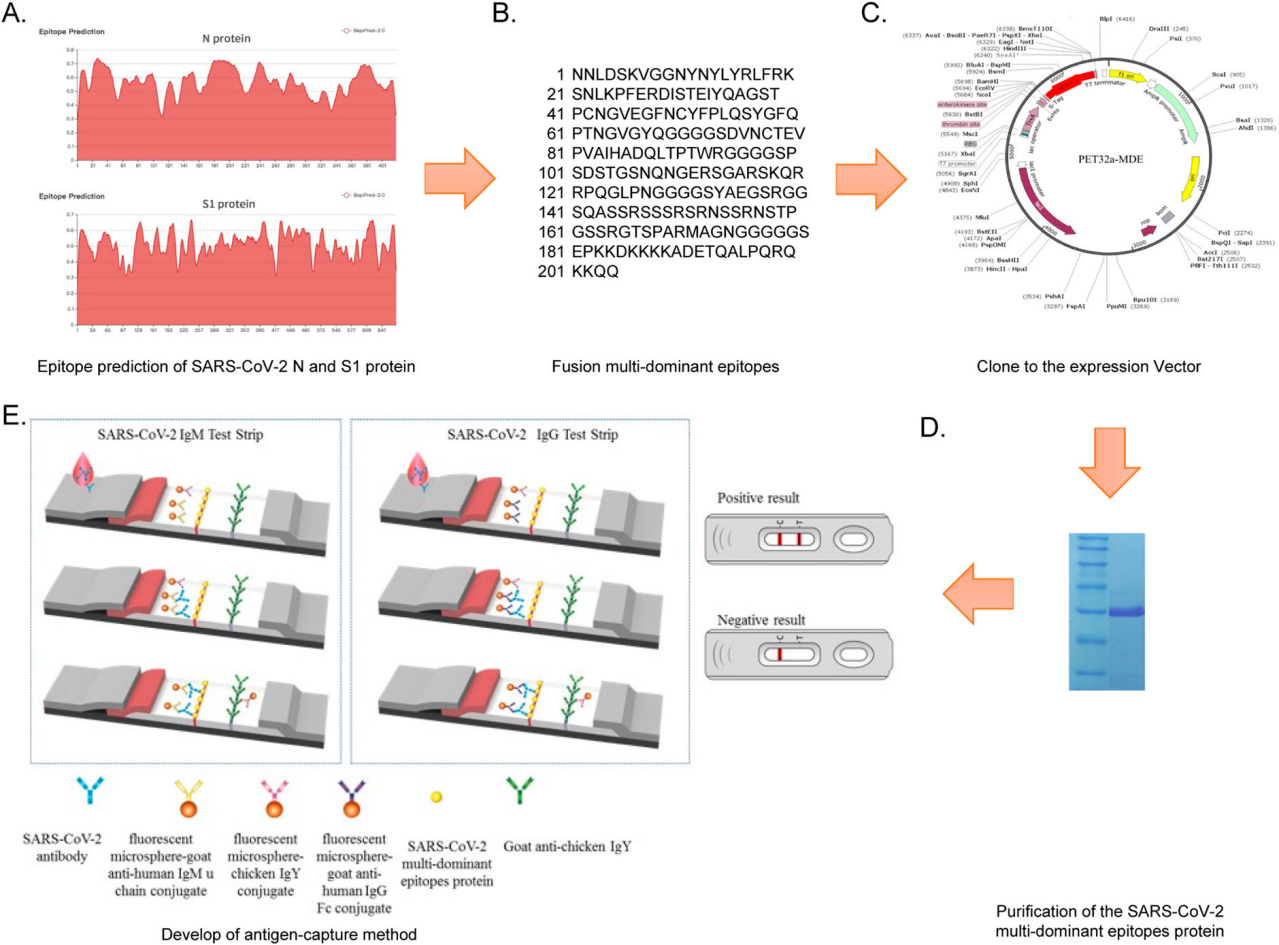
Operation of the lateral flow immunoassay. (A) Epitope prediction of the SARS-CoV-2 N and S1 protein. (B) Fusion multi-dominant epitopes. (C) Protein expression vector. (D) Purification of the SARS-CoV-2 multi-dominant epitopes protein. (E) Schematic representation of the assay's mechanism.

The dominant antigen epitopes of the SARS-CoV-2 full length N protein and S1 protein (mature form without Signal peptide) were predicted and screened through BepiPred-2.0 (Sequential B-Cell Epitope Predictor) (Figure 1(A), Table 1), and were connected by flexible peptides (GGGGS) to constitute the fusion recombinant protein of the SARS-CoV-2 multi-dominant epitopes (MDE) (Figure 1(B)). The sequence was synthesized by Beijing Tsingke Biological Technology, constructed to prokaryotic expression vector PET32a (Figure 1(C)), and transformed into *E. coli* BL21. The protein was induced to be expressed at a concentration of 1.0 mmol/L of IPTG, purified by Ni column affinity, and analysed by SDS-PAGE (Figure 1(D)).

**Table 1.** Epitope prediction of the N Protein and S1 protein using BepiPred-2.0.

Structural protein	Peptide No.	Sequence	Position
S1 protein	1	NNLDSKVGGNYNYLYRLFRKSNLKPFERDISTEIYQAGSTPCNGVEGFNCYFPLQSYGFQPTNGVGYQ	424–491
	2	DVNCTEVPVAIHADQLTPTWR	599–619
N protein	3	PSDSTGSNQNGERSGARSKQRRPQGLPN	20–47
	4	YAEGSRGGSQASSRSSSRSRNSSRNSTPGSSRGTSPARMAGNG	172–214
	5	EPKKDKKKKADETQALPQRQKKQQ	367–390

### Preparation of the goat anti-human IgG Fc antibody-time-resolved fluorescence microsphere conjugate, goat anti-human IgM µ chain antibody-time-resolved fluorescence microsphere conjugate, and chicken IgY antibody-time-resolved fluorescence microsphere conjugate

1. Activation: One-hundred μL of Time-resolved fluorescent microsphere was taken and mixed in 400 μL of activation buffer (100 mmol/L of MES pH 6.0), 2 mg of EDC was added, and left for oscillation and activation at room temperature for 30 min after being fully mixed.
2. Coupling: The suspension mentioned in (1) was abandoned from the supernatant after centrifugation at 10,000 rpm for 10 min at 4°C, and was re-suspended in 200 μL of coupling buffer (100 mmol/L of PB pH7.0) in triplicate. Then, 100 µg of goat anti-human IgG Fc antibody, goat anti-human IgM µ chain antibody, and chicken antibody were added, respectively, and were left for oscillation and coupling at 250°C for 30 min.
3. Sealing: The suspension mentioned in (2) was added to 20 μL of 1% BSA solution, and left for oscillation and sealing overnight at room temperature after being fully mixed.
4. Storage: The suspension mentioned in (3) was abandoned from the supernatant after centrifugation at 10,000 rpm for 10 min at 4°C, and re-suspended in storage buffer (PB buffer of 1% NaN_3_ and 1% BSA at pH 7.4). Then, microsphere was washed once by this method, and stored in the dark at 4°C after being fully mixed.

### Preparation of the glass fibre mats

A storage buffer was used to dilute the concentration of the goat anti-human IgG Fc antibody-time-resolved fluorescence microsphere conjugate and chicken IgY antibody-time-resolved fluorescence microsphere conjugate mixture (1:1 ratio), and the goat anti-human IgM µ chain antibody-time-resolved fluorescence microsphere conjugate and chicken IgY antibody-time-resolved fluorescence microsphere conjugate mixture (1:1 ratio) to 10 μg/mL. Then, this was sprayed on the glass fibre mat using the Bio-dot Spot-spray system, left to dry at 37°C for three hours, and collected for sealed storage.

**Table 2.** Statistical results of IgM and IgG in Healthy people and Non-COVID-19 disease patients (U/L).

Statistics	Healthy control		Non-COVID-19 Disease	
	IgM	IgG	IgM	IgG
Number of values	600	600	396	396
Minimum	0	0	0	0
25% Percentile	0.2705	0.2	0.11	0
Median	0.44	0.4	0.23	0.1
75% Percentile	0.64	0.6375	0.37	0.325
Maximum	5.73	2.43	3.8	1.79
5% Percentile	0.12	0.09	0	0
95% Percentile	1.08	1	0.734	0.593
Mean	0.5178	0.4585	0.2831	0.1911
Std. Error	0.01751	0.01333	0.0162	0.01165
Lower 95% CI of mean	0.4834	0.4323	0.2512	0.1682
Upper 95% CI of mean	0.5522	0.4847	0.3149	0.214

### Preparation of the nitrocellulose (NC) membrane

Next, 0.05 mol/L of PB buffer at pH 7.2 was used to dilute the fusion protein of the SARS-CoV-2 multi-dominant epitopes protein to 1 mg/mL, and the Bio-dot Spot-spray system was used to spray this to the test area (T) of the NC membrane at a quantity of 1.2 μL/cm. Then, 0.05 mol/L of PB buffer at pH 7.2 was used to dilute the goat-anti-chicken IgY antibody to 0.5 mg/mL. Afterwards, this was sprayed to the control area (C) of the NC membrane at a quantity of 1.2 μL/cm. Subsequently, this was left to dry at 37°C for 24 h for backup use.

### Assembly of the test strips

The sample absorption pad, glass fibre mat, NC membrane and water-absorbent pad were lapped, pasted and fixed on the bottom plate, from left to right, including the end of the sample absorption pad, and connected to the starting section of the glass fibre mat. Then, the end of the glass fibre mat was connected to starting section of the NC membrane, the end of the NC membrane was connected to the starting section of the water-absorbent pad, the starting section of the sample absorption pad was aligned with the starting section of the bottom plate, and the end of the water-absorbent pad was aligned with the end of the bottom plate. Afterwards, this was cut into small strips at a width of 3.96 mm using a machine, and the small strips were placed into tailored plastic cards. Thus, the test strips were made.

### Sample detection

Next, 10 μL of whole blood samples were added to 250 μL of the sample diluent and fully mixed. Then, 80 μL of the sample solution was pipetted to the sample well of the SARS-CoV-2 IgG antibody and IgM antibody test strip, respectively, and left at room temperature (20–25°C) for 15 min. Afterwards, the test strip cards were inserted into the carrier of the fluorescent detector, and the result was read.

## Data analysis and statistics

In order to determine how sensitive and specific the assay is, in the present primary experiment, 200 samples obtained from healthy controls were detected to determine the cut-off value. The 95% percentile of the T/C ratio (the ratio between the fluorescence intensity in test area [T] and the fluorescence intensity in control area [C] on test strip card) was defined as 1 U/L, and this was set as the cut-off value.

Statistics analysis was performed using the SPSS 20.0 software, and the nonparametric test and two-sided *X*^2^-test were used to compare the differences between the two groups. A *P*-value of <0.05 was considered statistically significant.

## Results

### Methodology evaluation of the analysis performance of the test strips

#### Lowest limit of detection (LLOD)

**Figure 2.**
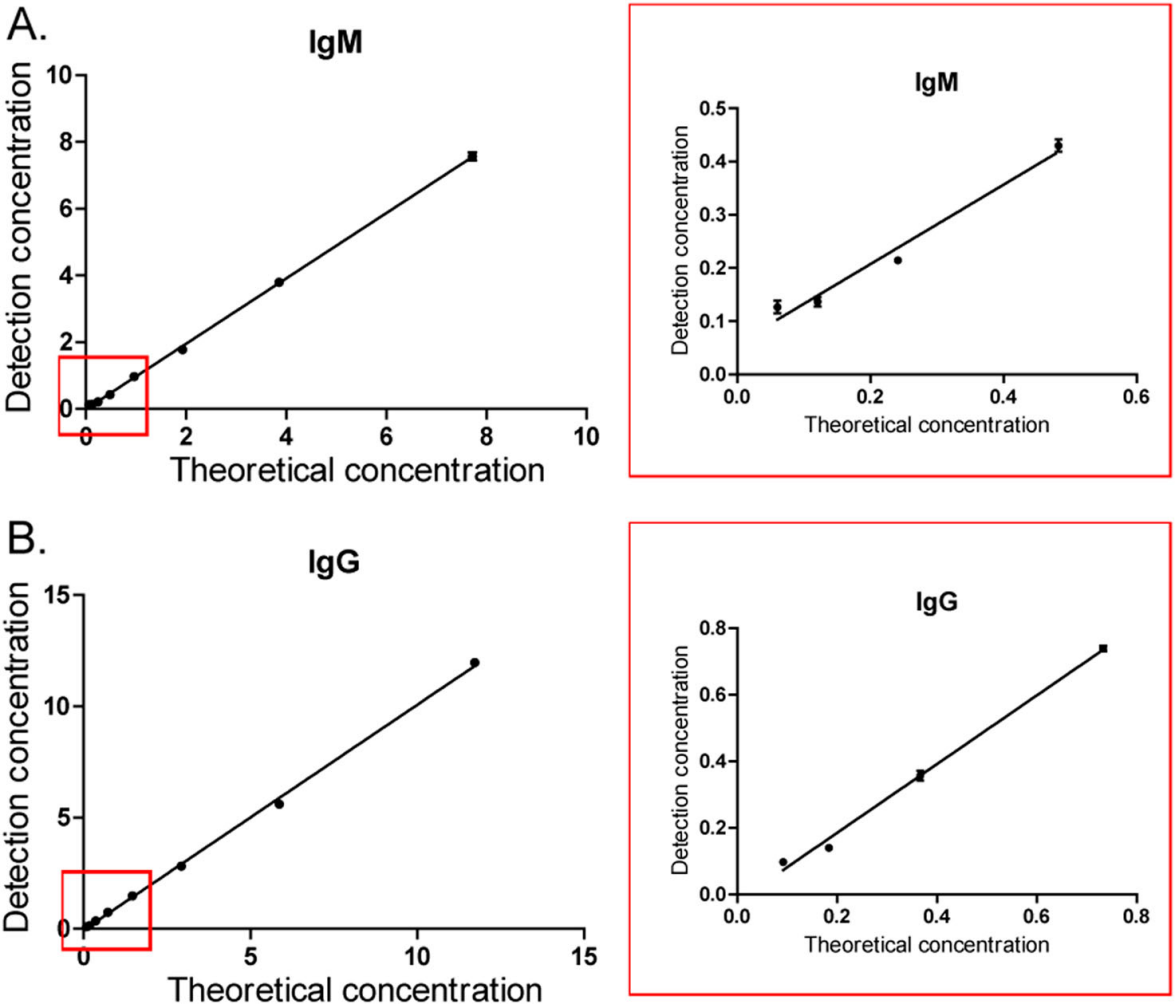
Lowest limit of detection of the test strip for IgM (A) and IgG (B).

The serum sample pool (SARS-CoV-2 IgM antibody, 38.6 U/L; SARS-CoV-2 IgG antibody, 58.6 U/L) was diluted for 5, 10, 20, 40, 80, 160, 320 and 640 times, until the result could not be tested. For the IgM antibody, the maximum dilution was 320 times, and the lowest limit of detection was 0.121 U/L. For the IgG antibody, the maximum dilution was 160 times, and the lowest limit of detection was 0.366 U/L (Figure 2).

#### Repeatability

Weak positive samples and strong positive samples were selected to measure the SARS-CoV-2 IgM and IgG tests for 10 times, respectively. The repeatability of the weak positive and strong positive samples was 6.5% (2.3 ± 0.15 U/L) and 8.7% (13.6 ± 1.18 U/L) for IgM, respectively, and 6.4% (3.3 ± 0.21 U/L) and 8.6% (23.1 ± 1.98 U/L) for IgG, respectively (Supplementary Table 1).

#### Cross reaction

The investigators tested the other antibodies using IgM/IgG strips. The results revealed that there was no cross reaction between these strips, and anti-influenza A (IgG/IgM), anti-influenza B (IgG/IgM), anti-229E (alpha coronavirus), anti-NL63 (alpha coronavirus), anti-OC43 (beta coronavirus), anti-HKU1 (beta coronavirus), and anti-respiratory syncytial virus (IgG/IgM) (Supplementary Table 2).

### Clinical sensitivity and specificity of the strips

**Figure 3.**
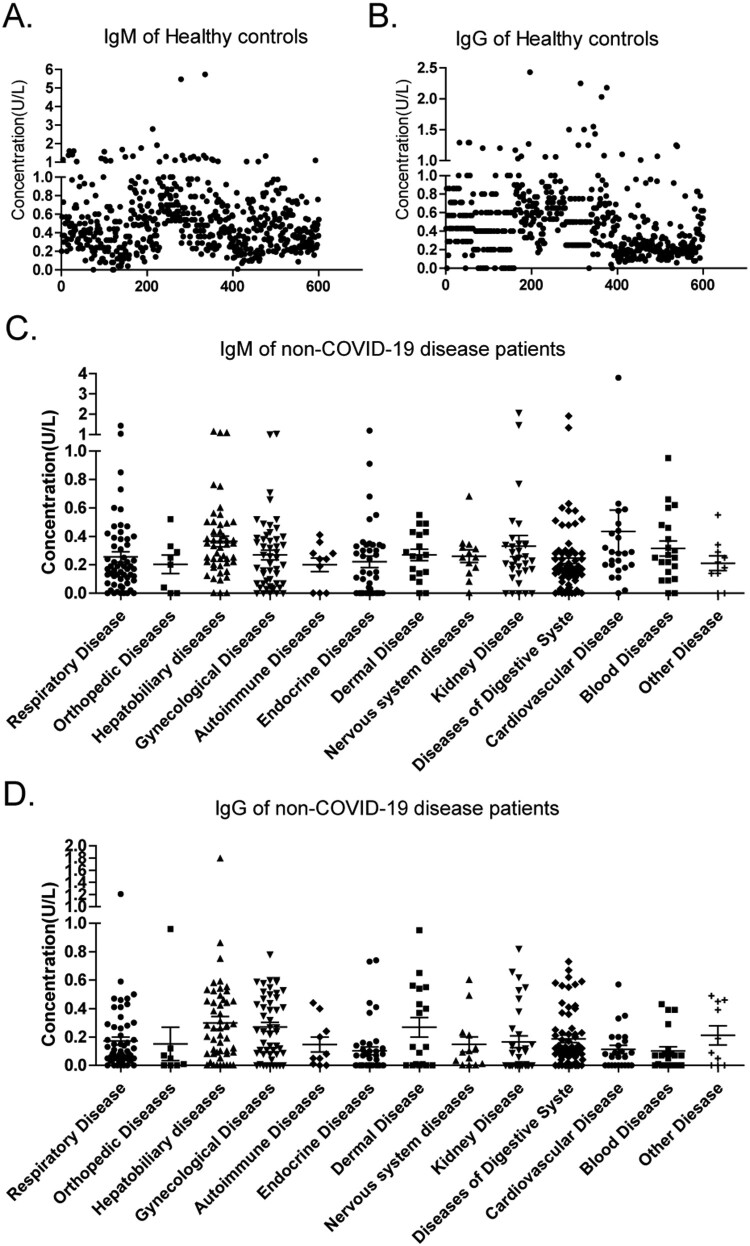
Clinical sensitivity and specificity. (A) IgM and IgG detection in healthy subjects, IgM (●) and IgG (▪). (B–C) IgM (B) and IgG (C) detection in patients with non-COVID-19 diseases.

The clinical samples obtained from 600 healthy controls (male, n = 313, female, n = 287; age range: 9–74, median age: 45) and 396 patients with other different diseases (male, n = 185, female, n = 211; age range: 1–94, median age: 50), and the 338 hospitalized samples obtained from 164 clinically confirmed COVID-19 patients (male, n = 92, female, n = 72; age range: 25∼91, median age: 62) and 234 follow-up COVID-19 patients (male, n = 115, female, n = 119; age range: 1–84, median age: 49) were selected to test the sensitivity and specificity of the new strips. Statistical results of IgM and IgG in healthy people and non-COVID-19 disease patients were shown in Table 2. As shown in Figure 3 and Table 3, the specificity of IgM and IgG was 94% and 95.50% in healthy people, respectively, and 96.72% and 99.49% in patients with non-COVID-19 disease, respectively. For patients undergoing treatment, the sensitivity of IgM and IgG differed among the three time periods: the first two weeks (<15 days), the third week (15–21 days), and the subsequent weeks (>21 days). As shown in Table 3, the sensitivity of the IgM strips during these three periods was 22.22%, 78.95% and 62.20%, respectively, and the sensitivity of the IgG strips during these three periods was 55.56%, 86.84% and 88.32%, respectively. For patients in the recovery stage, the sensitivity of IgM and IgG was 37.61% and 90.17%, respectively.

**Table 3.** Results of antibody detection in Healthy people and Non-COVID-19 disease.

Study group		No.tested		IgM positive	Sensitivity	Specificity (%)	IgG positive	Sensitivity	Specificity (%)
Healthy controls		600		36	-	94.00	27	-	95.50
Non-COVID-19 Disease		396		13		96.72	2		99.49
Respiratory disease		57		2	-	96.49	1	-	98.25
Orthopedic diseases		8		0	-	100.00	0	-	100.00
Hepatobiliary diseases		48		3	-	93.75	1	-	97.92
Gynecological diseases		50		2	-	96.00	0	-	100.00
Autoimmune diseases		10		0	-	100.00	0	-	100.00
Endocrine diseases		41		1	-	97.56	0	-	100.00
Dermal disease		18		0	-	100.00	0	-	100.00
Nervous system diseases		13		0	-	100.00	0	-	100.00
Kidney disease		32		2	-	93.75	0	-	100.00
Digestive disease		64		2	-	96.88	0	-	100.00
Cardiovascular disease		24		1	-	95.83	0	-	100.00
Blood diseases		21		0	-	100.00	0	-	100.00
Other disease (Neonatal diseases, oral diseases)		10		0	-	100.00	0	-	100.00
COVID-19 patients during treatment	338			213	63.02	-	295	87.28	-
The first two weeks (<15days)		9		2	22.22	-	5	55.56	-
The third week(15∼21days)		38		30	78.95	-	33	86.84	-
Three weeks later (>21days)		291		181	62.20	-	257	88.32	-
COVID-19 follow-up patients (>21days)			234	88	37.61	-	211	90.17	-
									

These results demonstrate that the IgM and IgG strips have high specificity, providing an efficiency approach for excluding SARS-CoV-2 infection in hospitals and isolated areas. IgM strips had a high detection rate in the early stage of infection, especially in the third week (15–21days). The IgG strips had a high sensitivity of over 88% in the later stage, both for patients undergoing treatment and cured patients.

### Dynamic monitoring of antibody IgG and IgM in the treatment stage of ordinary and severe COVID-19

**Figure 4.**
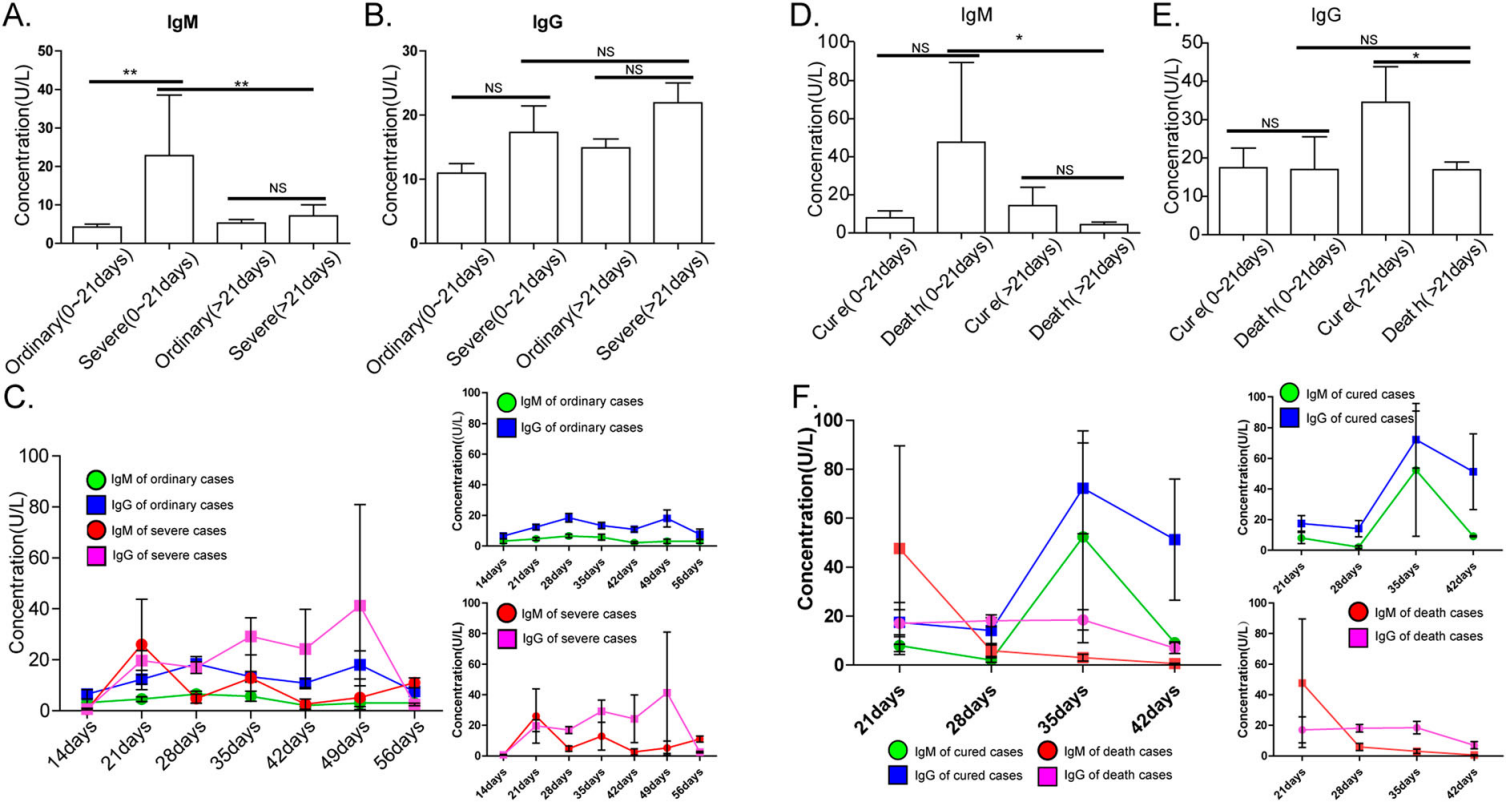
IgM and IgG detection in the process of COVID-19. (A–B) Comparison of IgM (A) and IgG (B) between severe and ordinary patients. NS, nonsense; **, *p *< 0.01. (C) Dynamic monitoring of antibody IgG and IgM in 93 COVID-19 patients during the treatment. Sera were collected from 0 to 70 days after the onset of symptoms. IgM in the ordinary group (green circle), IgG in the ordinary group (blue square), IgM in the severe group (red circle), and IgG in severe group (pink square). The error bars correspond to 1 S.D. (D-E) Comparison of IgM (A) and IgG (B) between cured cases and death cases in severe patients. NS, nonsense; *, *p *< 0.05. (F) Dynamic monitoring of antibody IgG and IgM in cured cases and death cases. IgM of n cured cases (green circle), IgG of cured cases (blue square), IgM of death cases (red circle), and IgG of death cases (pink square). The error bars correspond to 1 S.D.

The concentrations of IgM and IgG in the 94 clinically confirmed COVID-19 and hospitalized patients were dynamically collected and detected (3–5 days/test), with a total of 268 tests. Most of the hospitalized patients in the present study had COVID-19 characteristic manifestations, as determined by the CT images. Mild cases with no abnormal CT findings were treated in an isolated area. Therefore, samples obtained from hospitalized patients were categorized as ordinary cases (n = 141, No. tested = 280) and severe cases (n = 23, No. tested = 58) (severe cases + critically ill cases) based on *the Diagnosis and treatment of novel coronavirus pneumonia (Trial version 6).* As shown in Figure 4(A and B), the investigators compared the IgM and IgG concentrations between the groups of ordinary and severe cases (severe cases + critically ill cases) within the first three weeks (day 0∼21) and at three weeks (>day 21) after COVID-19 onset. In the first three weeks, the levels of IgM in the severe group were much higher than those in the ordinary group, indicating that severe patients had higher levels of immune response within the first three weeks. The results of the dynamic monitoring of IgM/IgG revealed that in the severe group, IgM and IgG alternately fluctuated in the development of COVID-19, with higher levels than those in the ordinary group (Figure 4(C)).

Since the present study is an observability experiment, the investigators further grouped these severe cases into cured cases (n = 10, No. tests = 19) and death cases (n = 15, No. tests = 39) based on the prognosis of patients. The results in Figure 4(D–F) revealed that levels of IgG in cured cases were higher than those in death cases (*p *< 0.05), and the continuous low levels of IgG after 28 days were associated with poor prognosis.

### Application of IgM and IgG test strips for the follow-up and suspected case screening

**Figure 5.**
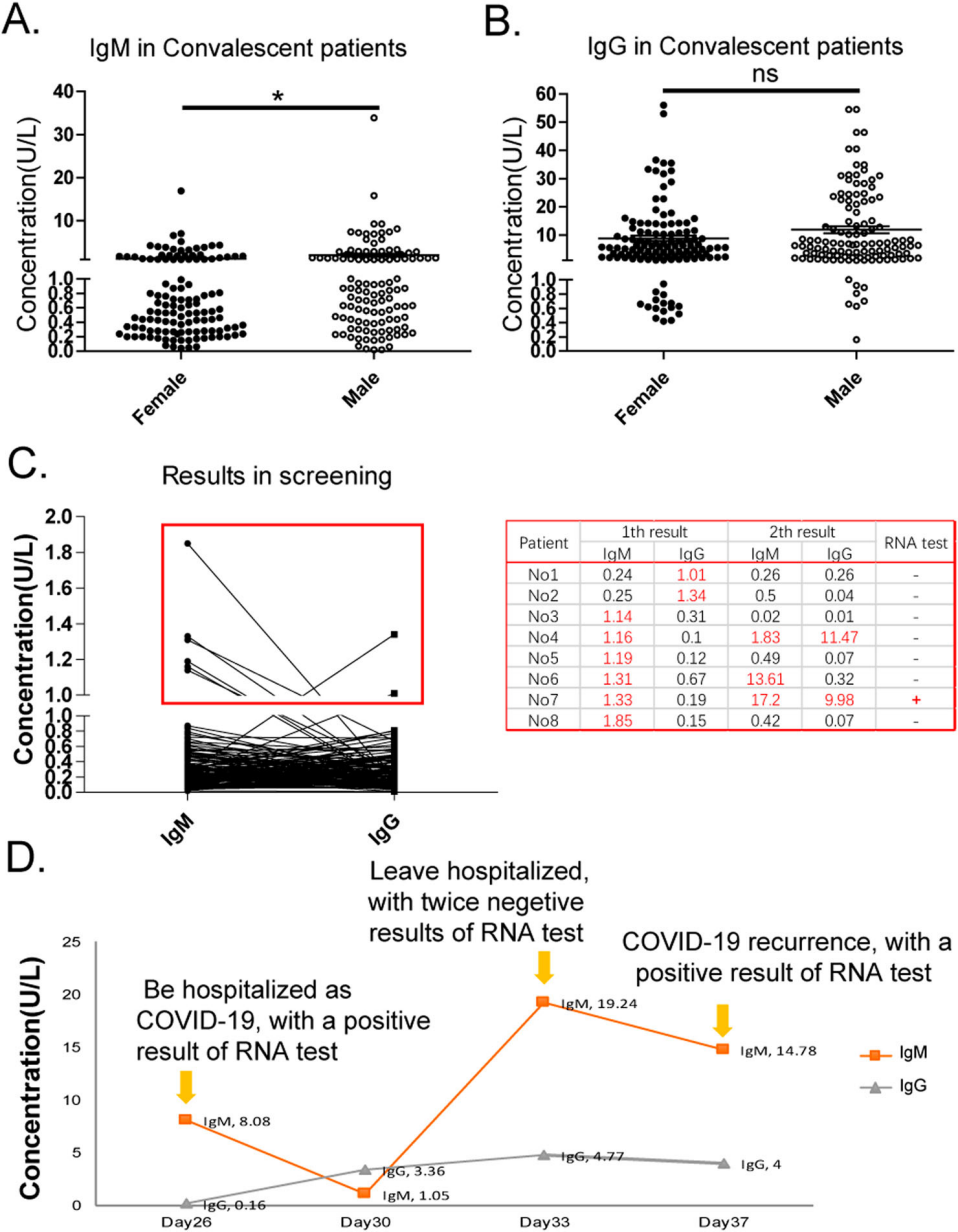
IgM and IgG levels in cured cases and death cases. (A–B) The result of the IgM (A) and IgG (B) detection in convalescent patients. Male (●), Female (○); ns, nonsense; *, *p *< 0.05. (C) The use of antibody detection in the suspected case screening. Cases in the red box were the monitored cases. (D) A case report.

A total of 234 patients who have had mild COVID-19 were followed up after treatment (>21 days), and received an antibody and RNA test. Among these patients, 100% of these patients had negative results in the RNA test, and 90.60% (212/234) of these patients had positive results in the antibody test, which included 37.61% (88/234) IgM positives and 90.61% (211/234) IgG positives. By observing the recent infection index IgM and long-term infection index IgG, it could be clearly determined whether these patients have successfully produced antibodies to the SARS-CoV-2 protein, or the body of these patients has established immune protection. The investigators explored the influence of gender and age factors on antibody titer, and found that the average level of IgM in convalescent patients was higher in males than in females (*p* = 0.0121, Figure 5(A)), but there was no statistical difference in age stratification (data not shown).

For the 154 suspected patients who had close contact with COVID-19 patients, the strips were used for screening and exclusion. Among these, eight patients (5.20%) had a week positive result in the antibody test, but simultaneously had a negative result in the RNA detection. The investigators continuously monitored these eight cases for 14 days, and it was found that three of these patients had elevated levels of IgM and IgG (Figure 5(C)). Finally, one of these three cases developed symptoms of fever, and was diagnosed with COVID-19 using the positive RNA test and characteristic CT image. The other patients (153/154) were eliminated, with no fever, negative result of RNA detection, and no abnormal in CT image. This result proves that antibody detection has the potential ability for suspected screening and disease prediction.

## Discussion

The present study is the first to describe the IgG and IgM antibody profiles of patients with COVID-19, in which serum samples were serially collected up to 0–70 days of onset of fever. A previous study [11] described that IgG and IgM can be positive before the clinical diagnosis, and that this may provide a quick, simple and accurate aided detection method for suspected COVID-19 patients, when compared to RNA detection. In a previous study in SARS, the level of IgG and IgM antibodies increased to detectable levels at the third week of illness [12]. Therefore, the investigators speculated that a similar phenomenon may occur in the course of the SARS-CoV-2 infection. In the present study, the recombinant protein and test strip for detecting the SARS-CoV-2 antibody by the antigen capturing method, and its preparation method were provided, supporting a new method for SARS-CoV-2 infection screening, diagnosis, disease monitoring and prognosis evaluation.

Compared to similar products, the brand-new methodology used in the present study was equipped with many advantages. Compared to the colloidal gold-based immunochromatographic assay, the strips realized the quantitative analysis and dynamic monitoring. Compared to the ELISA assay and chemiluminescence assay, the strips realized a low testing instrument cost, and a convenient (point-of-care test) and quick operation (within 15 min). Compared to nucleic acid detection, these strips had high specificity and sensitivity, which could be used as a supplement to nucleic acid detection, greatly improving the accuracy. Most importantly, the detection of IgM and IgG reflects the recent and long-term infection and immune response of the body.

This method can be applied to clinical practice and found to be useful for clinical diagnosis, monitoring, treatment and determining the prognosis of COVID-19. The virus-specific IgM and IgG in healthy subjects was 94% and 96.72%, respectively, while in non-COVID-19 disease patients, this was 95.50% and 99.49%, respectively. The sensitivity of IgM and IgG was evaluated in the COVID-19 treatment stage and recovery stage. In the treatment stage, the sensitivity of IgM in the first two weeks, the third week, and at three weeks was 22.22%, 78.95% and 62.20%, respectively, while the sensitivity of IgG in the three periods was 55.56%, 86.84% and 88.32%, respectively. In the recovery stage, the sensitivity of IgM and IgG was 37.61% and 90.17%, respectively. There were few false-positive and false-negative results (Table 3). Combined with the actual situation of detection, there were several reasons for the false negatives, such as detection time, individual differences, and low antibody concentration. The false-negative ratio of patients in severe conditions was higher than that in the ordinary group, suggesting that severe patients have a lower antibody response for SARS-CoV-2. Clinical practitioners analysed that the use of antibiotics, older age, more complications, and decreased immune function of body immunity may induce lower antibody levels and poor prognosis. Recent reports on COVID-19 infections have revealed that both community-dwelling older persons are at high risk [13]. The results of the dynamic monitoring of IgG and IgM revealed that in the severe group, IgM and IgG alternately fluctuated in the development of the disease, which was at a higher level, when compared to the ordinary group (Figure 4(C)). This may be due to the high tilter of the virus, the strong immune response, or the presence of other complications. Based on the different levels of IgM and IgG between cured cases and death cases in severe patients, the continuous low levels of antibody imply the loss of immune function and poor prognosis for the disease.

The case report in the present study shows that the virus-specific antibody detection can be used as a supplement to nucleic acid detection (Figure 5(D)). A hospitalized COVID-19 patient had a positive result in the RNA test on day 26 after the onset of fever. The results of the antibody test were, as follows: IgM 8.08 U/L (positive), IgG 0.21 U/L (negative). This suggested that the patient was still in the infection stage of SARS-CoV-2. On day 33, the patient was discharged with two negative results in the RNA test and a positive antibody test (IgM 19.24 U/L, IgG 4.77 U/L). Then, on day 37, the patient's COVID-19 recurrence had a positive result in the RNA test (IgM 14.78 U/L, IgG 4.00 U/L). This suggests that the antibody test is very important for the clinic judgement of the patient's recovery and discharge. In the present case, the continuous elevation of virus-specific IgM may indicate the development of COVID-19, and that it is not a good chance to discharge the patient.

For convalescent patients, observing the IgM and IgG is necessary for determining whether the patient has successfully established their body's immune defense to SARS-CoV-2. For close contact, the elevated level of IgM and IgG can not only predict the progress of the disease, but also indicate the existence of low concentration of activated immunity, but not pathogenicity (Figure 5(C)). On the basis of previous theories and research results for the close contact of HIV [14] and SARS [15], low dose virus stimulation may activate the immune system, but not develop the disease. Therefore, the titer in close contact may intimate a concentration reference range for live attenuated vaccines.

Now, the product “2019-nCoV IgG Antibody Determination Kit” and “2019-nCoV IgM Antibody Determination Kit” have been certified by CE (Registration number DE/CA22/419-1805-IVD). Due to the emergency of the outbreak of COVID-19, the investigators could not carry out long-term observations for COVID-19 patients, or distinguish the interference of other coronaviruses, such as SARS. Further studies and follow-ups would be conducted.

## Supplementary Material

Supplementary_data.docx

## Data Availability

All data generated or analysed during this study are included in this published article.
