# Supplementary material for: Establishing a high sensitivity detection method for SARS-CoV-2 IgM/IgG and developing a clinical application of this method
Source: Emerg Microbes Infect. 2020 Sep 18;9(1):2020–9. doi: 10.1080/22221751.2020.1811161 (PMC7534335; doi:10.1080/22221751.2020.1811161)
Supplement: Supplementary_data.docx [file TEMI_A_1811161_SM8678.docx]

**Supplementary data**

**Supplementary Table 1. Repeatability**

| Test results  (U/L) | IgM strips | |  | IgG strips | |
| --- | --- | --- | --- | --- | --- |
|  | Weak positive sample | Strong positive sample |  | Weak positive sample | Strong positive sample |
| 1 | 2.31 | 12.5 |  | 3.11 | 27.85 |
| 2 | 2.34 | 12.8 |  | 3.34 | 21.8 |
| 3 | 2.51 | 13.4 |  | 3.51 | 21.8 |
| 4 | 2.3 | 12.1 |  | 3.5 | 23.1 |
| 5 | 2.2 | 14.6 |  | 3.2 | 21.6 |
| 6 | 2.52 | 13.4 |  | 3.52 | 23.4 |
| 7 | 2.28 | 13.1 |  | 3.72 | 23.1 |
| 8 | 2.1 | 15.1 |  | 3.1 | 22.6 |
| 9 | 2.32 | 13.4 |  | 3.32 | 21.2 |
| 10 | 2.6 | 15.8 |  | 3.12 | 24.9 |
| Mean | 2.35 | 13.62 |  | 3.34 | 23.14 |
| SD | 0.15 | 1.18 |  | 0.21 | 1.98 |
| CV(%） | 6.54 | 8.67 |  | 6.38 | 8.57 |

**Supplementary Table 2. Cross-reaction test results**

| Object | Detection Results | Judgment Results |
| --- | --- | --- |
| Anti-influenza A (IgG/IgM) | 0.4±0.1 | Negative |
| Anti-influenza B (IgG/IgM) | 0.2±0.1 | Negative |
| Anti-229E (alpha coronavirus) | 0.05±0.01 | Negative |
| Anti-NL63 (alpha coronavirus) | 0.52±0.05 | Negative |
| Anti-OC43 (beta coronavirus) | 0.42±0.11 | Negative |
| Anti-HKU1 (beta coronavirus) | 0.6±0.15 | Negative |
| Anti-respiratory syncytial virus (IgG/IgM) | 0 | Negative |
